# Hydrothermal-assisted exfoliation of Y/Tb/Eu ternary layered rare-earth hydroxides into tens of micron-sized unilamellar nanosheets for highly oriented and color-tunable nano-phosphor films

**DOI:** 10.1186/s11671-015-0828-0

**Published:** 2015-03-14

**Authors:** Qi Zhu, Zhixin Xu, Ji-Guang Li, Xiaodong Li, Yang Qi, Xudong Sun

**Affiliations:** Key Laboratory for Anisotropy and Texture of Materials (Ministry of Education), School of Materials and Metallurgy, Northeastern University, No. 3-11, Wenhua Road, Shenyang, Liaoning 110819 China; Advanced Materials Processing Unit, National Institute for Materials Science, Namiki 1-1, Tsukuba, Ibaraki 305-0044 Japan; Institute of Materials Physics and Chemistry, School of Sciences, Northeastern University, No. 3-11, Wenhua Road, Shenyang, Liaoning 110819 China

**Keywords:** Layered rare-earth hydroxide, Ultra-large unilamellar nanosheets, Hydrothermal anion exchange, Oriented films, Color-tunable emission

## Abstract

Efficient exfoliation of well-crystallized (Y_0.96_Tb_*x*_Eu_0.04-*x*_)_2_(OH)_5_NO_3_ · *n*H_2_O (0 ≤ *x* ≤ 0.04) layered rare-earth hydroxide (LRH) crystals into tens of micron-sized unilamellar nanosheets has been successfully achieved by inserting water insoluble oleate anions (C_17_H_33_COO^−^) into the interlayer of the LRH via hydrothermal anion exchange at 120°C, followed by delaminating in toluene. The intercalation of oleate anions led to extremely expanded interlayer distances (up to approximately 5.2 nm) of the LRH crystals and accordingly disordered stacking of the *ab* planes along the *c*-axis and also weakened interlayer interactions, without significantly damaging the *ab* plane. As a consequence, the thickness of the LRH crystals increased from approximately 1 to 10 μm, exhibiting a behavior similar to that observed from the smectite clay in water. Highly [111]-oriented and approximately 100-nm thick oxide films of **(**Y_0.96_Tb_*x*_Eu_0.04-*x*_**)**_2_O_3_ (0 ≤ *x* ≤ 0.04) have been obtained through spin-coating of the exfoliated colloidal nanosheets on quartz substrate, followed by annealing at 800°C. Upon UV excitation at 266 nm, the oxide transparent films exhibit bright luminescence, with the color-tunable emission from red to orange, yellow, and then green by increasing the Tb^3+^ content from *x* = 0 to 0.04.

**Electronic supplementary material:**

The online version of this article (doi:10.1186/s11671-015-0828-0) contains supplementary material, which is available to authorized users.

## Background

Layered inorganic compounds have interesting physical/chemical properties, such as tunable interlayer spacing and interlayer composition, and can be readily functionalized via intercalation to produce specific properties [1]. In addition, they may potentially be delaminated into unilamellar nanosheets or nanosheets of few-layer thick via ion exchange, followed by mechanical agitation in a proper medium [1]. The obtained nanosheets can serve as ideal building blocks for the construction of inorganic or hybrid organic–inorganic multifunctional films owing to their significantly two-dimensional morphologies (lateral size up to microns and thickness down to nanometer level) [2-6]. Because of the significant morphological anisotropy, the nanosheets tend to orient themselves, with a certain crystallographic direction perpendicular to substrate surface, and thus introduce additional or greatly enhanced functionalities. Delaminating layered compounds into nanosheets attracted much attention, and monolayer nanosheets have been successfully exfoliated from several types of layered inorganic materials, such as layered double hydroxides (LDHs) [7,8], graphite [9], metal oxides [10], phosphates [11], and chalcogenides [12].

Layered rare-earth hydroxides (LRHs) [13-26], with a general formula of RE_2_(OH)_5_(A^*m*-^)_1/*m*_ · *n*H_2_O (rare-earth (RE) ions; intercalated (A) anions), are a new group of important anion-type layered materials that may potentially be exfoliated into single or few-layer thick nanosheets for the further construction of various nanostructures, particularly transparent functional films. Due to the unique electronic, optical, magnetic, and catalytic properties of the rare-earth elements, LRHs attracted immediate attentions for controllable synthesis since their emergence, and some efforts have been paid to the thinning of LRHs via exfoliation [22,23,26] and exfoliation-free synthesis [24]. Recently, LRHs crystals have been exfoliated into nanosheets by several research groups via anion exchange with dodecylsulfate (DS^−^) at room temperature, followed by mechanical agitation in formamide [22,23]. Despite these successes, the high-charge density of LRHs makes a complete exfoliation rather challenging. Previous studies also showed that exfoliation usually takes several days and is thus an arduous and lengthy work [22]. We have obtained in our previous work ultra-thin LRHs nanosheets (down to approximately 4 nm), without exfoliation, by capping thickness growth of the crystals with tetrabutylammonium ions (TBA^+^, (C_4_H_9_)_4_ N^+^) in hydrothermal reaction, but the nanosheets are limited to submicron in lateral size [24]. It is also worth noting that the LRHs particles synthesized through the current techniques are mostly platy crystals of several micrometers in lateral dimension, so the final exfoliated nanosheets are submicron sized [22]. Very recently, unilamellar nanosheets with lateral sizes ≥60 μm and thicknesses of only approximately 1.6 nm have been efficiently delaminated by us from sub-millimeter-sized LRHs crystals [25] via hydrothermal anion exchange of the interlayer NO_3_^−^ with dodecylsulfate (C_12_H_25_OSO_3_^−^, DS^−^), followed by exfoliation in formamide. Significantly faster anion exchange and higher extent of DS^−^ intercalation were observed for the hydrothermal than ambient processing [26].

The interlayer space of LRH is significantly affected by the size of the intercalated anions, and a more weakened interlayer interaction via insertion of bigger anions is beneficial to exfoliation. Water-soluble dodecylsulfate (C_12_H_25_OSO_3_^−^, DS^−^), which has a long carbon chain, is usually employed to swell anion-type layered compounds for delamination via room temperature anion exchange, and successes were manifested in the cases of LDHs [7,8] and LRHs [22]. Anions of even longer carbon chain, such as oleate (C_17_H_33_COO^−^), would be more efficient for interlayer expansion but are hardly soluble in water at room temperature. We show in this work the successful insertion of oleate anions into the interlayers of tens of micron-sized LRH crystals via hydrothermal anion exchange and based on which the efficient exfoliation of ultra-large (approximately 20 μm) and single layer (approximately 1.55 nm) nanosheets in toluene. Highly [111]-oriented oxide films have also been constructed via self-assembly of the resultant nanosheets for multi-color emissions. In the following sections, we report the hydrothermal intercalation of oleate into LRHs crystals of the Y/Tb/Eu ternary system, exfoliation of nanosheets, and assembly of transparent films with the nanosheets for color-tunable emissions. The materials are characterized in detail by the combined techniques of field emission scanning electron microscopy (FE-SEM), transmission electron microscopy (TEM), X-ray diffraction (XRD), Fourier transform infrared spectroscopy (FTIR), atomic force microscopy (AFM), and optical spectroscopy, and we believe that the outcomes of this work would have wide implications to other layered inorganic materials.

## Methods

### Synthesis

The starting rare-earth sources for LRH synthesis are Y_2_O_3_, Tb_4_O_7_, and Eu_2_O_3_, all 99.99% pure products from Huizhou Ruier Rare-Chem. Hi-Tech. Co. Ltd (Huizhou, China). Analytical grade nitric acid (HNO_3_, 63 wt.%), ammonium hydroxide solution (NH_3_ · H_2_O, 25 wt.%), and ammonium nitrate (NH_4_NO_3_, 99.0% pure) were purchased from Shenyang Chemical Regent Factory (Shenyang, China). The nitrate solution of RE^3+^ was prepared by dissolving the corresponding oxide with a slightly excessive amount of nitric acid, followed by evaporation at approximately 90°C to dryness to remove the superfluous acid. Synthesis of ultra-large LRH crystals for the Y/Tb/Eu ternary system was conducted via hydrothermal reaction (180°C for 24 h) in the presence of NH_4_NO_3_, as described in our previous paper [25]. The optimal concentration of either Tb^3+^ or Eu^3+^ in Y_2_O_3_ is approximately 4 to 5 at.%, above which concentration quenching of luminescence would take place. This value would hold for the Tb^3+^/Eu^3+^ pair, and thus the total concentration of Tb^3+^ and Eu^3+^ is fixed at 4 at.% in this work.

### Hydrothermal-assisted anion exchange, exfoliation, and film construction

For a typical anion-exchange reaction, 0.4 mmol of (Y_0.96_Tb_*x*_Eu_0.04-*x*_)_2_(OH)_5_NO_3_ · *n*H_2_O (0 ≤ *x* ≤ 0.04) was dispersed in 50 mL of water containing a proper amount of sodium oleate (C_17_H_33_COONa). The resultant suspension was transferred into a Teflon lined stainless-steel autoclave of 100 mL capacity after being stirred for 5 min. The autoclave was tightly sealed and was put in an electric oven preheated to 120°C. After 24 h of reaction, the autoclave was left to cool naturally to room temperature, and the anion-exchange product was collected via centrifugation. The wet precipitate was washed with hot distilled water (80°C) for three times, rinsed with absolute ethanol, and was finally dried in air at 50°C for 24 h. The anion-exchange product was then dispersed in 50 mL of toluene, and a transparent colloidal suspension was obtained after constant magnetic stirring for 12 h. The resultant nanosheets were assembled into films on quartz substrates (10 mm in diameter) via spin-coating. Briefly, 200 μL of the transparent colloidal suspension was dropped on the substrate fixed on a spin coater, spun at 2,000 revolutions per minute (rpm) for 1 min to assemble the nanosheets, followed by slow air drying. Prior to spin coating, the quartz substrate was ultrasonically cleaned in sequence in acetone, ethanol, and distilled water, and was then immersed in a mixed solution (3:1 in volume ratio) of H_2_SO_4_ (30 vol.%) and H_2_O_2_ (30 vol.%) heated to 80°C for 1 h. Subsequently, the substrate was kept in the mixed solution of H_2_O:NH_4_OH:30 vol.% H_2_O_2_ (5:1:1 in volume ratio) to render surface hydrophilicity. Before use, the substrate was immersed in an aqueous solution of polyethylenimine (PEI, 1.5 mg/mL), soaked in a poly sodium 4-styrene sulfonate (PSS) aqueous solution (1 mg/mL) for 1 h, followed by washing with distilled water for three times and drying. Oxide film was obtained by calcining the LRH film in flowing oxygen (200 mL/min) at 800°C for 4 h, followed by reducing in flowing hydrogen (200 mL/min) at 800°C for 2 h for the Tb^3+^ containing samples.

### Characterization techniques

Phase identification was performed by XRD (Model PW3040/60, Philips, Eindhoven, the Netherlands) operating at 40 kV/40 mA using nickel-filtered Cu *K*α radiation and a scanning speed of 4.0° 2*θ*/min. Lattice constants were calculated from the XRD patterns using the software package X’Pert HighScore Plus version 2.0 (PANanalytical B.V., Almelo, the Netherlands). Morphologies of the products were observed via FE-SEM (Model JSM-7001 F, JEOL, Tokyo, Japan) and TEM (Model JEM-2000FX, JEOL, Tokyo). FTIR (Model Spectrum RXI, Perkin-Elmer, Shelton, CT, USA) of the pristine and anion-exchanged LRHs was performed by the standard KBr method. Chemical composition of the products was determined via elemental analysis for the Y/Tb/Eu content by the inductively coupled plasma spectrophotometric method with an accuracy of 0.01 wt.% (ICP, Model IRIS Advantage, Nippon Jarrell-Ash Co. Ltd., Kyoto, Japan), for NO_3_^−^ via spectrophotometry (Ubest-35, Japan Spectroscopic Co., Ltd., Tokyo, Japan), and for the carbon content on a simultaneous carbon/sulfur determinator with a detection limit of 0.01 wt.% (Model CS-444LS, LECO, St. Joseph, MI, USA). A Nanosurf easyScan 2 AFM (Nanosurf, Liestal, Switzerland) was employed to obtain topographical images of the nanosheets. Optical properties of the phosphor films were measured at room temperature with a UV–vis spectrophotometer (Lambda-750S, Perkin-Elmer, Shelton, CT, USA) for transmittance and with an LS-55 fluorescence spectrophotometer (Perkin-Elmer, Shelton, CT, USA) for photoluminescence excitation (PLE) and emission (PL).

## Results and discussion

**Figure 1:**
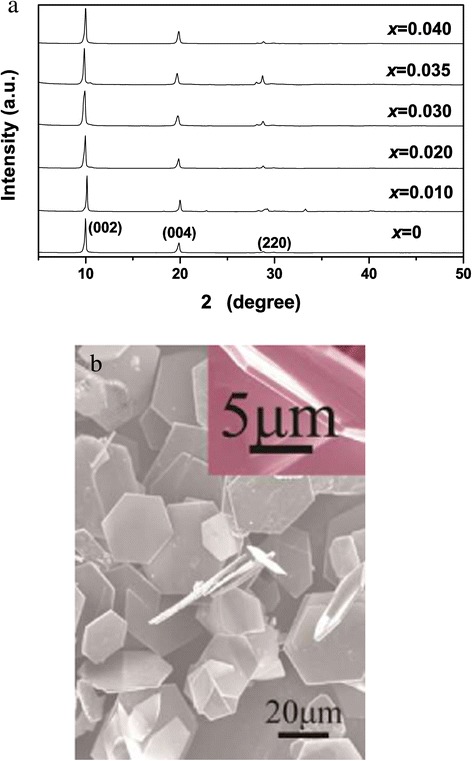
**XRD patterns of the hydrothermal products and FE-SEM morphology of the** ***x*** **= 0.035 sample. (a)** XRD patterns of the (Y_0.96_Tb_*x*_Eu_0.04-*x*_)_2_(OH)_5_NO_3_ · *n*H_2_O ( 0 ≤ *x* ≤ 0.04) LRH solid solutions hydrothermally synthesized at 180°C and **(b)** FE-SEM morphology of the *x* = 0.035 sample.

Well-crystallized and ultra-large LRHs crystals can be synthesized by autoclaving mixed nitrate solution of the component rare-earths at 180°C to 200°C and in the presence of NH_4_NO_3_ mineralizer [25]. Figure 1a shows XRD patterns of the hydrothermal products with various amounts of Tb^3+^. A series of strong (00 *l*) reflections were observed to be characteristic of a layered phase, as previously reported for the Ln_2_(OH)_5_NO_3_ · *n*H_2_O LRHs (see Additional file 1: Table S1) [13-21]. A number of weak non-(00 *l*) reflections were also detected, indicating that the LRHs are ordered for their hydroxide main layers. The solid solutions were found to have similar lattice constants of *a* ~ 1.273, *b* ~ 0.715, and *c* ~ 1.800 nm, due to the small total content (4 at.%) of Tb^3+^ and Eu^3+^ and the similar sizes of the two kinds of ions (for eightfold coordination, ${r}_{T{b}^{3+}} = 0.1040\ \mathrm{nm}$ and ${r}_{E{u}^{3+}} = 0.1066\ \mathrm{nm}$). Figure 1b shows FE-SEM morphology of the *x* = 0.035 sample, where the crystals were observed to be uniform hexagons of ≥30 μm in lateral size and approximately 1 μm in thickness. The straight and sharp crystal edges with intersection angles of approximately 120° may suggest high crystallinity of the platelets. Similar morphologies were observed for the other Y/Tb/Eu combinations and are thus not shown.

**Figure 2:**
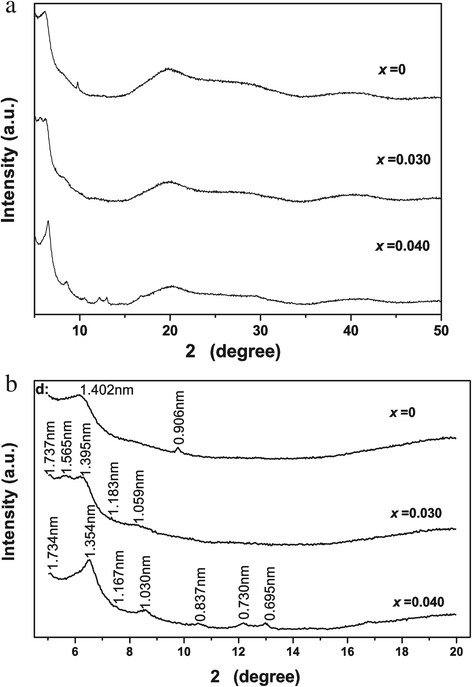
**XRD patterns of the LRH-oleate samples obtained by hydrothermal anion exchange. (b)** is part of **(a)** in the 2*θ* range of 4.5° to 20.5°.

Figure 2 shows XRD patterns of the anion exchange products (denoted as LRH-oleate hereafter) obtained via hydrothermal reaction at 120°C for 24 h, from which it is seen that the non-(00 *l*) reflections, such as (220), disappeared from the LRH-oleate, though the (00 *l*) reflections are still observable. Several sets of (00 *l*) reflections were observed for each of the products, suggesting the existence of multi interlayer distances as found in our previous work [26]. For the *x* = 0.040 sample (Figure 2b), the sharp and symmetric (00 *l*) reflections at 1.734, 1.030, and 0.730 nm indicate a basal spacing of approximately 5.15 nm, those at 1.354 and 0.837 nm correspond to a basal spacing of approximately 4.10 nm, and the ones at 1.167 and 0.695 nm conform to a basal spacing of approximately 3.50 nm. Similarly, the *x* = 0.030 sample has basal spacings of approximately 5.20, 4.70, 4.20, and approximately 3.50 nm, and the *x* = 0.00 sample has spacing values of approximately 4.50 and 4.20 nm. The existence of multi-spacings suggests that the oleate anions may have moved into the interlayers of the LRHs via the ‘wriggle intercalation’ model proposed recently [26]. It was noted that the maximum basal spacing of approximately 5.20 nm is much larger than that of the LRH-DS^−^ (approximately 3.70 nm) under identical hydrothermal anion-exchange [22,26], revealing the higher efficiency of oleate over DS^−^ in LRH swelling. In addition, quasi-amorphous diffractions were observed in the 2*θ* ≥ 15° range, different from the LRH-DS^−^ obtained with the same hydrothermal treatment. In the latter case, immobile (220) and other non-(00 *l*) reflections are clearly observable [22,26]. Since the oleate anion (C_17_H_33_COO^−^) has a longer carbon chain than DS^−^ (C_12_H_25_OSO_3_^−^), diffusion of a large amount of oleate into the interlayer gallery may have resulted in the higher degrees of swelling observed from Figure 2, similar to the ‘osmotic hydration’ of smectite clay in water and the interlayer expansion of LDH-DS^−^ in formamide. The extremely expanded interlayer may disorder the stacking of *ab* planes in the [001] direction, and thus amorphous XRD diffractions appeared. The *ab* plane, however, was not significantly damaged, as inferred from the appearances of (00 *l*) reflections. The extremely swollen LRH-oleate with long-range ordered *ab* planes would be beneficial to exfoliation.

**Figure 3:**
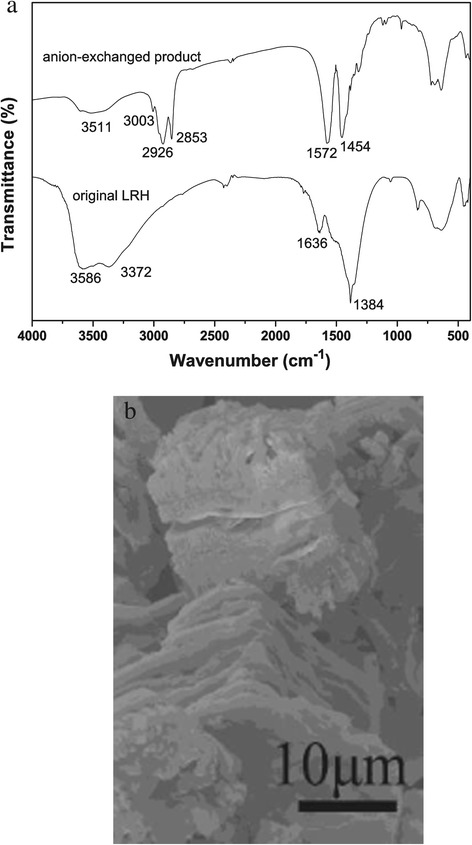
**FTIR spectra for the pristine LRH and LRH-oleate and the typical morphology of LRH-oleate. (a)** FTIR spectra for the pristine LRH (*x* = 0.035) and its oleate-exchange derivative. **(b)** FE-SEM morphology of the LRH-oleate sample. Similar results were observed for the rest samples studied in this work.

Figure 3a shows FTIR spectra for the pristine LRH and LRH-oleate. For LRH, the absorption peaks at approximately 3,372 cm^−1^ and the shallow shoulder near 1,636 cm^−1^ provide evidence for water of hydration in the structure, and they are assignable to the O-H stretching vibrations (ν_1_ and ν_3_) and the H-O-H bending mode (ν_2_), respectively [27,28]. The absorption band observed in the range of 3,500 to 3,750 cm^−1^ (centered at approximately 3,586 cm^−1^) is indicative of hydroxyl (OH^−^) groups [27,28]. The strong absorption peak at 1,384 cm^−1^ is characteristic of an uncoordinated nitrate anion, as also found for other layered hydroxides containing free interlayer nitrate groups [13,21,27,28]. After anion exchange, the vibration of nitrate is no longer observable. Instead, two intense bands appeared at approximately 1,572 and 1,454 cm^−1^, which are assignable to the stretching modes of carboxyl (COO^−^) [27,28]. The strong absorptions at approximately 2,926 and 2,853 cm^−1^ are due to the asymmetric and symmetric CH_2_ stretching vibrations, respectively, whereas the weak band at approximately 3,003 cm^−1^ is assignable to the stretching mode of the terminal CH_3_ group of the hydrocarbon tail [27,28]. The above results confirmed a complete replacement of the interlayer nitrate by oleate. Chemical analysis yielded a general formula of Ln_2_(OH)_5_(C_17_H_33_COO)(C_17_H_33_COOH)_*y*_ · *n*H_2_O (Ln = Y, Tb, Eu) by applying molecular neutrality, assuming that all the C are from C_17_H_33_COO^−^ and C_17_H_33_COOH (Additional file 1: Table S2). The results of chemical analysis comply with the FTIR observations. Figure 3b shows the typical morphology of LRH-oleate, from which it is seen that the thickness of LRH platelets has been significantly expanded from approximately 1 to 10 μm, and, owing to the massive insertion of oleate anions, cracks of different gaps are formed along the thickness direction.

**Figure 4:**
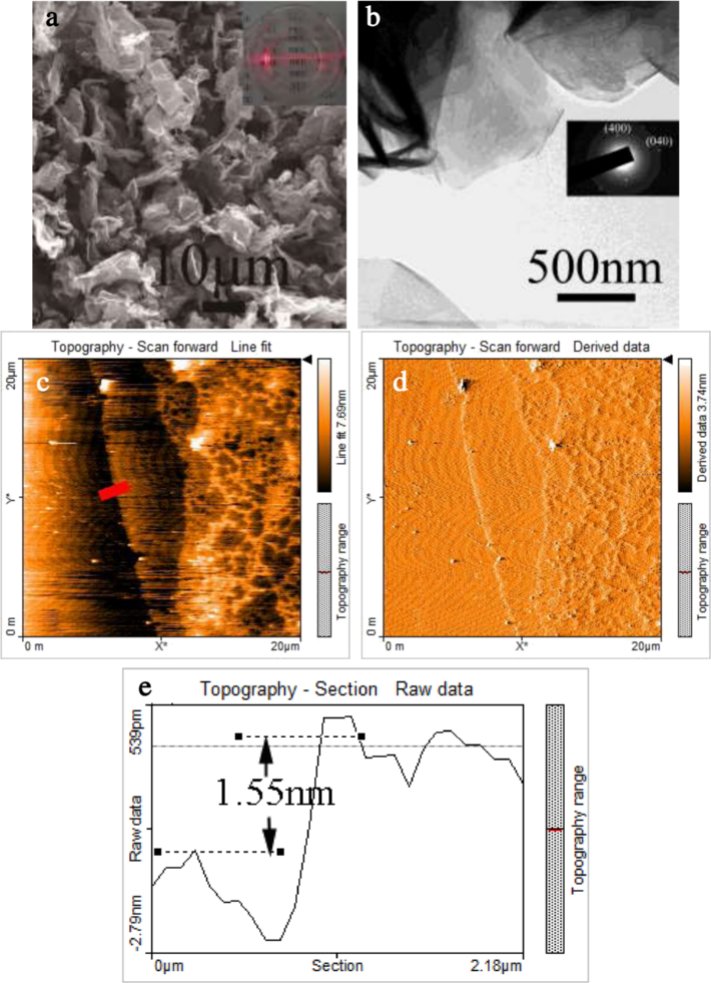
**FE-SEM, TEM, AFM images and height profile of the exfoliated nanosheets. (a)** FE-SEM and **(b)** TEM micrographs showing morphologies of the nanosheets exfoliated from LRH-oleate (*x* = 0.035). The AFM images **(c,d)** and the height profile **(e)** along the red line marked in **(c)**, respectively. The inset in **(a)** shows the appearance of a colloidal suspension of the nanosheets in toluene, with a clearly observable Tyndall effect under laser beam irradiation. The inset in **(b)** is the SAED pattern of an individual unilamellar nanosheet.

Dispersing the LRH-oleate in 50 mL of toluene yielded a transparent colloidal suspension via constant and slow magnetic stirring for 12 h. The clearly observable Tyndall effect under laser beam irradiation (inset in Figure 4a) indicates the delamination of LRH-oleate. FE-SEM observation found that most of the exfoliated nanosheets have lateral sizes of ≥20 μm (Figure 4a). The uniform contrast under TEM (Figure 4b) of the individual nanosheets implies that the nanosheet is rather thin. Selected area electron diffraction (SAED) yielded well-arranged spot-like patterns, suggesting that the nanosheet under observation is well crystallized and is of single crystalline (inset in Figure 4b). The cell parameters calculated from the SAED pattern are *a* ~ 1.27 and *b* ~ 0.72 nm, in good agreement with those of the bulk LRH [21]. The nanosheet was estimated to be approximately 1.55 nm thick from the AFM height profile (Figure 4e), indicating that the nanosheet is primarily of unilamellar. At the same time, AFM observation indicated that the nanosheets are very flat and smooth (Figures 4c,d). Possibly due to surface chemical adsorption of oleate and toluene molecules, the unilamellar nanosheets are a little thicker than the crystallographic thickness of 0.93 nm [24]. Compared with those reported previously [22,23], the unilamellar nanosheets obtained in this work showed a significantly larger lateral size and a more unabridged shape, which is advantageous for the construction of highly oriented functional films.

**Figure 5:**
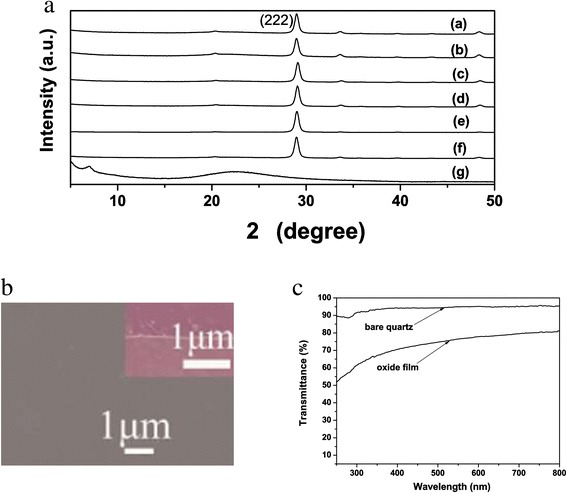
**XRD patterns, FE-SEM image, and transmission spectrum of the films. (a)** XRD patterns of the (Y_0.96_Tb_*x*_Eu_0.04-*x*_)_2_O_3_ (0 ≤ *x* ≤ 0.04) oxide films (lines a-f) calcined from the LRH films constructed with exfoliated nanosheets, where (a)-(f) correspond to *x* = 0, 0.01, 0.02, 0.03, 0.035, and 0.04, respectively. Line (g) in **(a)** is for the LRH nanosheet film (*x* = 0.035). **(b)** and **(c)** are FE-SEM image and transmission spectrum of the **(**Y_0.96_ Tb_0.035_Eu_0.005_ **)**_2_O_3_ film, respectively. The inset in **(b)** is the cross-section view of the film.

Depositing 200 μL colloidal nanosheets (solid loading: approximately 2 vol.%) on a quartz substrate followed by spin-coating has produced highly *c*-axis-oriented films via self-assembly of the nanosheets (Figure 5a(g)). As there are oleate anions and toluene molecules on surfaces of the positively charged nanosheets, the nanosheets tend to assemble themselves into new layered materials similar to LRH, and thus the (00 *l*) reflection was observed from the LRH film in Figure 5a(g). Calcining the LRH films at 800°C for 4 h, followed by hydrogen reduction at the same temperature for 2 h for the Tb-containing ones, yielded cubic-structured **(**Y_0.96_Tb_*x*_Eu_0.04-*x*_**)**_2_O_3_ (0 ≤ *x* ≤ 0.04) films (Figure 5a(a-f)). Because the projection in the [001] direction for the LRHs crystal and in the [111] direction for the cubic oxide crystal present close similarities in terms of rare-earth atomic configuration, the phase transformation from LRH to oxide is a quasitopotactic one [24]. The oxide films are thus highly [111] oriented and show strong (222) while very weak non-(222) reflections. Calculation with the (222) diffraction yielded similar cell constants of approximately 1.0664 nm for all the oxide films, similarly due to the small total content of Tb^3+^ and Eu^3+^. The oxide films are flat and significantly denser than those made with submicron-sized nanosheets [24], showing the great advantages of larger sheet size (Figure 5b). The oxide films were estimated to be approximately 100 nm thick via cross-section FE-SEM view (the inset in Figure 5b), and exhibit high transmittances of ≥75% (bare quartz: approximately 94%, Figure 5c) in the visible wavelength region (500 to 800 nm).

Although the macroscopic concentration of Tb^3+^/Eu^3+^ activators is fixed at 4 at.% in this work, concentration difference exists among different crystal planes. The (222) facet of Y_2_O_3_ is a close-packed one and thus has higher Y^3+^ occupancy. As the activators randomly replace Y^3+^, it can thus be said that more activators would reside on (222) to yield significantly enhanced emission. As shown in our previous work [24,26], the [111]-oriented (Y, Eu)_2_O_3_ film exhibited an emission intensity ≥2 times that of the randomly oriented powder of the same composition. Therefore, the highly [111]-oriented **(**Y_0.96_Tb_*x*_Eu_0.04-*x*_**)**_2_O_3_ (0 ≤ *x* ≤ 0.04) films fabricated in this work are expected to yield bright emissions.

(Additional file 1: Figure S1) shows PLE/PL spectra of the highly [111]-oriented (Y_0.96_Eu_0.04_)_2_O_3_ (*x* = 0) and (Y_0.96_ Tb_0.04_)_2_O_3_ (*x* = 0.04) films. For (Y_0.96_Eu_0.04_)_2_O_3_, the excitation spectrum consists of a broad and intense band at around 240 nm, which can be assigned to the charge-transfer (CT) from O^2−^ to Eu^3+^ [29,30]. Upon UV excitation at 240 nm, the oxide film exhibits sharp lines ranging from 500 to 700 nm, which are associated with the transitions from the excited ^5^D_0_ to the ^7^F_*J*_ (*J* = 0,1,2,3) ground states of Eu^3+^ [29,30]. Relative intensity of the transition to different *J* levels depends on the site symmetry of Eu^3+^, and the dominant red emission at 613 nm arises from the hypersensitive ^5^D_0_ → ^7^ F_2_ forced electric dipole transition of Eu^3+^ taking the non-centrosymmetric C_2_ lattice sites. The (Y_0.96_ Tb_0.04_)_2_O_3_ film exhibits a broad and strong excitation band in the 250- to 330-nm region with a maximum at 276 nm corresponding to the well-documented 4f^8^ → 4f^7^5d^1^ Tb^3+^ transition [31]. When excited at 276 nm, the oxide film displayed the typical ^5^D_4_ − ^7^F_*J*_ (*J* = 5 to 2) transitions of Tb^3+^ at about 543, 600, 627, and 671 nm, respectively, with the strongest emission being at 543 nm for green [31]. Overlapping the red- and green-emitting films yielded a bright yellow color under 254-nm radiation from a hand-held UV lamp (right part of Additional file 1: Figure S1), indicating that the emission color can be tuned by varying the Tb/Eu molar ratio. The excitation spectra of (Y_0.96_Eu_0.04_)_2_O_3_ (*x* = 0) and (Y_0.96_ Tb_0.04_)_2_O_3_ (*x* = 0.04) intersect at 266 nm, which would thus represent the most efficient wavelength to simultaneously excite Eu^3+^ and Tb^3+^.

**Figure 6:**
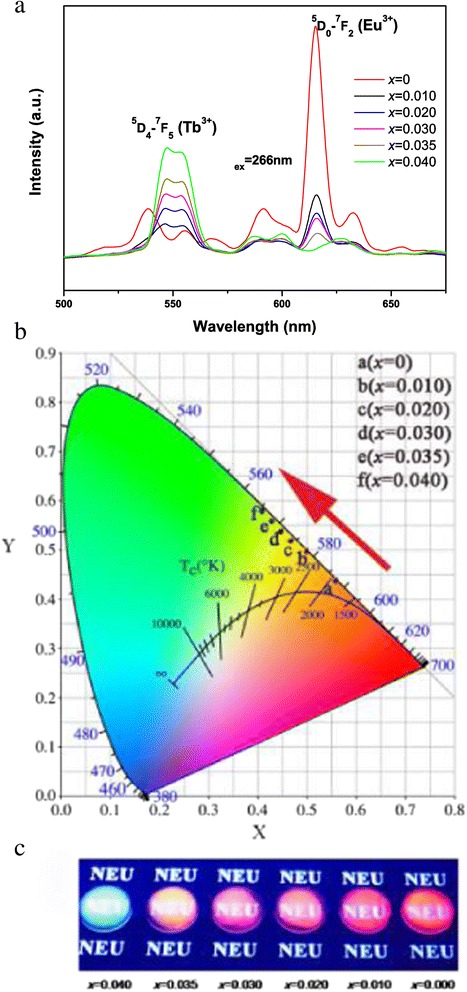
**Photoluminescence spectra, CIE chromaticity diagram, and multi-color emission of the oxide films.** Photoluminescence spectra **(a)** and CIE chromaticity diagram **(b)** of the (Y_0.96_Tb_*x*_Eu_0.04-*x*_)_2_O_3_ (0 ≤ *x* ≤ 0.04) films. Part **(c)** shows multi-color emission of the oxide films under 254-nm irradiation from a hand-held UV lamp.

**Figure 7:**
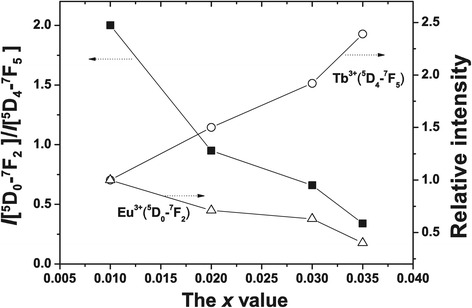
**The relative intensities of two typical emission peaks.** Correlation of the relative intensities *I*(^5^D_0_ → ^7^ F_2_) and *I*(^5^D_4_ → ^7^ F_5_) and the *I*(^5^D_0_ → ^7^ F_2_)/*I*(^5^D_4_ → ^7^ F_5_) intensity ratio with Tb content for (Y_0.96_Tb_*x*_Eu_0.04-*x*_)_2_O_3_. The relative intensities are obtained by normalizing the observed PL intensities to that of the *x* = 0.01 sample.

Photoluminescence (PL) of the **(**Y_0.96_Tb_*x*_Eu_0.04-*x*_**)**_2_O_3_ films (0 ≤ *x* ≤ 0.04) were systematically investigated to define the emission behavior and emission color (Figure 6a). It is seen that the Eu^3+^ emission at approximately 613 nm monotonically decreases while the Tb^3+^ emission at 543 nm improves with increasing Tb incorporation (Figures 6a and 7). We analyzed in Figure 7 the relative intensities of these two peaks together with the *I*(^5^D_0_ → ^7^ F_2_)/*I*(^5^D_4_ → ^7^ F_5_) intensity ratio as a function of the Tb content (0.010 ≤ *x* ≤ 0.035). Clearly, the ratio decreases from approximately 2.0 at *x* = 0.010 to approximately 0.34 at *x* = 0.035, suggesting color-tunable emissions. When excited at 266 nm, the samples have Commission Internationable Ed I’eclairage (CIE) chromaticity coordinates of (0.56, 0.44) for *x* = 0, (0.50, 0.50) for *x* = 0.010, (0.47, 0.52) for *x* = 0.020, (0.45, 0.54) for *x* = 0.030, (0.43, 0.56) for *x* = 0.035, and (0.41, 0.58) for *x* = 0.040 (Figure 6b). All these emission colors fall into the red to green region of the CIE chromaticity diagram, and the films exhibit bright colors changing from red to orange, yellow, and then green as shown in Figure 6c. Tb^3+^ → Eu^3+^ energy transfer (ET) is well known in the phosphors codoped with Tb^3+^/Eu^3+^ because of the substantial spectral overlap between the ^5^D_4_ → ^7^F_*J*_ emissions of Tb^3+^ and the ^7^ F_0,1_ → ^5^D_0–2_ excitation absorptions of Eu^3+^, and the efficiency of ET can be analyzed when the Tb^3+^ content is fixed [31]. Similar analysis, however, can hardly be made in this work since both the Eu^3+^ and Tb^3+^ contents are variables.

## Conclusions

In this work, tens of micron-sized unilamellar nanosheets were efficiently exfoliated from well-crystallized (Y_0.96_Tb_*x*_Eu_0.04-*x*_)_2_(OH)_5_NO_3_ · *n*H_2_O (0 ≤ *x* ≤ 0.04) layered rare-earth hydroxides (LRHs) via hydrothermal anion exchange of the interlayer NO_3_^−^ with much larger oleate anions, which were then employed for the construction of oriented fluorescent films. Detailed characterizations of the products by the combined techniques of FE-SEM, TEM, XRD, FT-IR, AFM, and PLE/PL have yielded the following main conclusions:

1. Inserting water insoluble oleate anions (C_17_H_33_COO^−^) into the interlayer of LRHs can be successfully achieved via hydrothermal processing (120°C for 24 h), which disorders the stacking of the *ab* plane along the *c* direction and weakens the interaction between the adjacent layers but with little damage to the *ab* plane. The intercalation of oleate extremely expands the interlayer distance up to approximately 5.2 nm, resulting in thickness increase of the LRH crystals from approximately 1 to 10 μm.
2. Delamination of the oleate-inserted LRHs into unilamellar nanosheets with lateral sizes of ≥20 μm and a thickness of approximately 1.55 nm has been achieved by dispersing LRH-oleate in toluene, followed by slow stirring.
3. Highly [111]-oriented and transparent films of **(**Y_0.96_Tb_*x*_Eu_0.04-*x*_**)**_2_O_3_ (0 ≤ *x* ≤ 0.04), with thicknesses of approximately 100 nm, have been constructed through spin-coating the colloidal nanosheets on quartz substrates, followed by calcination at 800°C. Upon UV excitation at 266 nm, the oxide films exhibit bright emissions and emission color can be tuned from red, orange, yellow, and then to green by increasing the Tb^3+^ content.

## Additional file

Additional file 1:
**Chemical analysis for LRHs and LRH-oleate and PL/PLE spectra and color emissions of two typical oxide films.** **Table S1.** The results of chemical analysis and the derived chemical formula for the pristine LRHs. **Table S2.** The results of chemical analysis and the derived chemical formula for the LRH-oleate. **Figure S1.** Photoluminescence excitation and emission spectra of the (Y_0.96_Tb_*x*_Eu_0.04-*x*_)_2_O_3_ (*x* = 0 and 0.04) films and the appearance of luminescence under 254-nm radiation from a hand-held UV lamp.
