# Supplementary material for: Hydrothermal-assisted exfoliation of Y/Tb/Eu ternary layered rare-earth hydroxides into tens of micron-sized unilamellar nanosheets for highly oriented and color-tunable nano-phosphor films
Source: Nanoscale Res Lett. 2015 Mar 14;10:132. doi: 10.1186/s11671-015-0828-0 (PMC4385236; doi:10.1186/s11671-015-0828-0)
Supplement: Additional file 1: — Chemical analysis for LRHs and LRH-oleate and PL/PLE spectra and color emissions of two typical oxide films. Table S1. The results of chemical analysis and the derived chemical formula for the pristine LRHs. Table S2. The results of chemical analysis and the derived chemical formula for the LRH-oleate. Figure S1. Photoluminescence excitation and emission spectra of the (Y0.96TbxEu0.04-x)2O3 (x = 0 and 0.04) films and the appearance of luminescence under 254-nm radiation from a hand-held UV lamp. [file 11671_2015_828_MOESM1_ESM.pdf]

Additional file

Hydrothermal-assisted exfoliation of Y/Tb/Eu ternary layered rare-earth hydroxides into tens of micron sized unilamellar nanosheets for highly oriented and color-tunable nano-phosphor films

Qi Zhu[1], Zhixin Xu[1], Ji-Guang Li[1,2*], Xiaodong Li[1], Yang Qi[3] and Xudong Sun[1]

[1]*Key Laboratory for Anisotropy and Texture of Materials (Ministry of Education), School of Materials and Metallurgy, Northeastern University, Shenyang, Liaoning 110819, China*

[2]*Advanced Materials Processing Unit, National Institute for Materials Science, Namiki 1-1, Tsukuba, Ibaraki 305-0044, Japan*

[3]*Institute of Materials Physics and Chemistry, School of Sciences, Northeastern University, Shenyang, Liaoning 110819, China*

**E-mail: LI.Jiguang@nims.go.jp*

Table S1. The results of chemical analysis for the pristine LRH and the derived chemical formula. Carbon was assumed to solely come from CO_3^{2-} and CO_3^{2-} was assumed to replace OH^-. The amount of OH^- was derived from molecular neutrality.

Sample ID	Chemical analysis (wt%)					Chemical Formula
	Y	Tb	Eu	NO_3^-	C	
S1	47.6	0	3.29	17.3	0.27	$(Y_{0.961}Eu_{0.039})_2(OH)_{4.84}(NO_3)_{1.0}(CO_3)_{0.08}\cdot 1.52H_2O$
S2	47.9	0.89	2.29	17.2	0.20	$(Y_{0.963}Tb_{0.010}Eu_{0.027})_2(OH)_{4.89}(NO_3)_{0.99}(CO_3)_{0.06}\cdot 1.49H_2O$
S3	47.6	1.86	1.61	17.6	0.30	$(Y_{0.960}Tb_{0.021}Eu_{0.019})_2(OH)_{4.80}(NO_3)_{1.02}(CO_3)_{0.09}\cdot 1.40H_2O$
S4	47.6	2.48	0.93	16.9	0.13	$(Y_{0.961}Tb_{0.028}Eu_{0.011})_2(OH)_{4.94}(NO_3)_{0.98}(CO_3)_{0.04}\cdot 1.60H_2O$
S5	47.6	3.01	0.34	17.2	0.17	$(Y_{0.962}Tb_{0.034}Eu_{0.004})_2(OH)_{4.90}(NO_3)_{1.0}(CO_3)_{0.05}\cdot 1.56H_2O$
S6	47.5	3.72	0	16.9	0.27	$(Y_{0.958}Tb_{0.042})_2(OH)_{4.86}(NO_3)_{0.98}(CO_3)_{0.08}\cdot 1.50H_2O$

Table S2. The results of chemical analysis for LRH-oleate and the derived chemical formula.

Sample ID	Chemical analysis (wt%)				Chemical Formula
	Y	Tb	Eu	C	
S1’	20.3	0	1.17	45.5	$(Y_{0.965}Eu_{0.035})_2(OH)_5(C_{17}H_{33}COO)(C_{17}H_{33}COOH)_{0.85}\cdot 2.82H_2O$
S2’	21.8	0.44	0.89	45.4	$(Y_{0.965}Tb_{0.011}Eu_{0.024})_2(OH)_5(C_{17}H_{33}COO)(C_{17}H_{33}COOH)_{0.72}\cdot 2.86H_2O$
S3’	22.2	0.82	0.63	48.3	$(Y_{0.965}Tb_{0.019}Eu_{0.016})_2(OH)_5(C_{17}H_{33}COO)(C_{17}H_{33}COOH)_{0.80}\cdot 2.22H_2O$
S4’	23.6	1.26	0.51	46.9	$(Y_{0.959}Tb_{0.029}Eu_{0.012})_2(OH)_5(C_{17}H_{33}COO)(C_{17}H_{33}COOH)_{0.64}\cdot 2.11H_2O$
S5’	23.5	1.60	0.21	45.2	$(Y_{0.958}Tb_{0.037}Eu_{0.005})_2(OH)_5(C_{17}H_{33}COO)(C_{17}H_{33}COOH)_{0.59}\cdot 2.12H_2O$
S6’	22.1	1.96	0	53.1	$(Y_{0.953}Tb_{0.047})_2(OH)_5(C_{17}H_{33}COO)(C_{17}H_{33}COOH)_{1.09}\cdot 1.71H_2O$

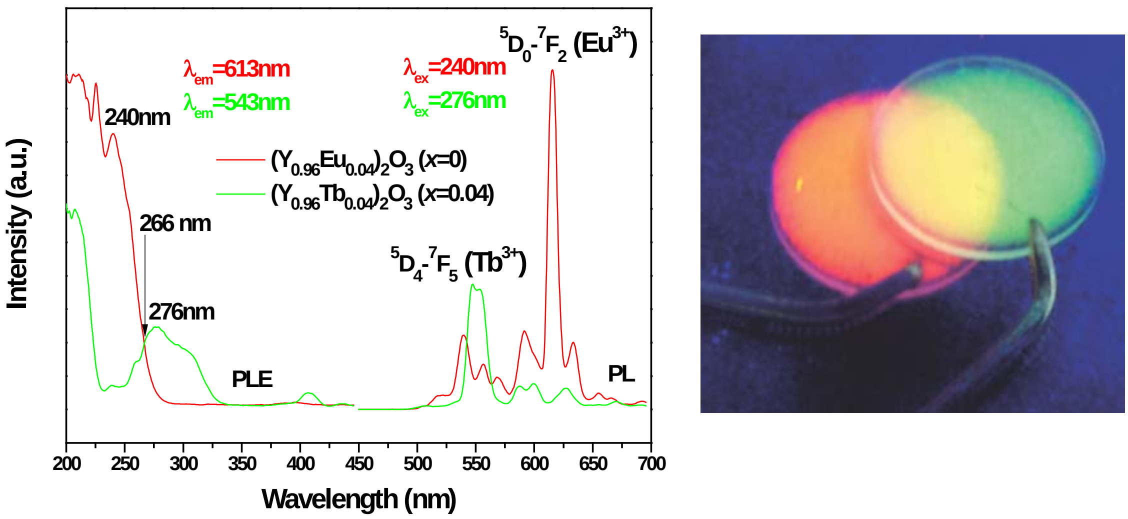

Figure S1. Photoluminescence excitation and emission spectra of the $(Y_{0.96}Tb_xEu_{0.04-x})_2O_3$ (x=0 and 0.04) films (left) and the appearance of luminescence under 254 nm radiation from a hand-held UV lamp (right).
